# Supplementary material for: Multi-omic comparative analysis of COVID-19 and bacterial sepsis-induced ARDS
Source: PLoS Pathog. 2022 Sep 19;18(9):e1010819. doi: 10.1371/journal.ppat.1010819 (PMC9484674; doi:10.1371/journal.ppat.1010819)
Supplement: Attachment — Submitted filename: response_letter.pdf [file ppat.1010819.s009.pdf]

Response letter

Original reviewer comments are shown in black font. Responses in are blue font.

Part I – Summary

Please use this section to discuss strengths/weaknesses of study, novelty/significance, general execution and scholarship.

Reviewer #1:

The study by Batra et.al. compares the multi-omic landscape of COVID-19-induced versus sepsis-induced ARDS. The study is descriptive, and in my opinion is well done with an appropriate acknowledge of limitations. I have few questions to ask for clarification:

Reviewer #2:

The authors performed a multi-omic comparative analysis of COVID-19 and bacterial sepsis-induced ARDS. They profiled plasma samples using metabolomics, lipodomics, and proteomics. Firstly, after comparing the molecular profiles between COVID-19 ARDS and bacterial sepsis-induced ARDS, they identified 706 differentially regulated molecules and 40 biological processes. They found that the overactivation of arginine metabolism involved in long-term sequelae of ARDS and that JAK inhibitors may improve outcomes in bacterial sepsis-induced ARDS. Secondly, they compared molecular associations with clinical manifestation to obtain an overview of the similarities and differences in molecular presentation of severity in both groups. They found that mitochondrial dysregulation might lead to post-ARDS renal-sequalae, and there was a synergy between prothrombotic processes, namely IL-17, MAPK, TNF signaling pathways, and cell adhesion molecules.
In general, this is a multi-omics study investigating the molecular characterization of differences between two ARDS etiologies. The interpretation of results and the conclusions are appropriate, and the work is important and can provide useful information. It has the potential for novel therapeutic development. However, there are a few issues to be addressed.

Reviewer #3:

The team led by Jan Krumsiek (strong researcher in this field) delivers an interesting multi-omics comparative analysis of COVID-19 and bacterial sepsis-induced ARDS.

(i) They identified 706 molecules differently abundant between the two ARDS etiologies, revealing more than 40 biological processes differently regulated between the two groups and assembled a cascade of therapeutically relevant pathways downstream of sphingosine metabolism.
(ii) The analysis suggests a possible overactivation of arginine metabolism involved in long-term sequelae of ARDS and highlights the potential of JAK inhibitors to improve

outcomes in bacterial sepsis-induced ARDS.
(iii) The second part of our study involved the comparison of the two ARDS groups with respect to clinical manifestations. Using a data-driven multi-omic network, we identified signatures of acute kidney injury (AKI) and thrombocytosis within each ARDS group.
(iv) The AKI-associated network implicated mitochondrial dysregulation which might lead to post-ARDS renal-sequalae.
(v) The thrombocytosis-associated network hinted at a synergy between prothrombotic processes, namely IL-17, MAPK, TNF signaling pathways, and cell adhesion molecules.

--> A combination therapy targeting two or more of these processes may ameliorate thrombocytosis-mediated hypercoagulation.

Response: We thank the reviewers for the valuable comments and appreciation of our work. We hope that we were able to address their concerns satisfactorily during the revision (see below).

Part II – Major Issues: Key Experiments Required for Acceptance

Please use this section to detail the key new experiments or modifications of existing experiments that should be absolutely required to validate study conclusions.

Generally, there should be no more than 3 such required experiments or major modifications for a "Major Revision" recommendation. If more than 3 experiments are necessary to validate the study conclusions, then you are encouraged to recommend "Reject".

Reviewer #1:

1) COVID-19 ARDS has a SOFA score of 10 and a mortality of 26%, which looks rather low for this type of severity

Response: This cohort was collected at the beginning of the pandemic (Mar/Apr 2020). Outcomes in this cohort are comparable to the outcomes observed in other academic centers at the time. For instance, Massachusetts General Hospital (1) and Mayo (2) had an early mortality rate of 17% and 11 % in their ICU units.

2) Additionally, the plateau pressure of sepsis ARDS is 20 with a quite low peep, and a very low P/F ratio. There is some disconnect there, can the authors confirm that the classification is correct? What is the ventilatory mode of these patients? Is that uniform? My feeling is that there is heterogeneity of vent settings, which could be a limitation that needs to be addressed as well.

Response: We agree with the reviewer that the plateau pressure and PEEP are low for the resultant P/F ratio. We confirmed that this data was correct and noted that the ventilatory modes were a mix of pressure-controlled and volume-controlled ventilation. Thus, there was heterogeneity in the settings which may represent a limitation. We have added the following sentence to the manuscript section 2.1

"Further, relatively low plateau pressure and extrinsic PEEP in the bacterial sepsis-induced ARDS group suggest that heterogeneous ventilator strategies were used in this population, which may affect the generalization of some findings from that group."

3) When were the blood samples collected? At hospital admission? Later?

Response: Samples were collected after admission. We have added this information in the methods section 4.3.

"Samples were collected after ICU admission. For bacterial-sepsis ARDS samples, the median was 1.5 days after admission, with an interquartile range: 1.0-2.0, and for COVID-19 ARDS, the median was 6 days with an interquartile range 3.5-9.5."

4) Can the authors explain the medications patients received at the moment of sampling? That could highly confound the omic results.

Response: Medication information was available for only 4 bacterial ARDS patients. Thus, the effect of medication on omics profiles could not be assessed.

Medication data were more comprehensibly available for COVID-19 ARDS patients. To evaluate possible influences of these medications on omics profiles, a linear stepwise backward selection approach was used (Toledo et al(3)) using medications administered on at least 4 samples. No significant effect of these medications was identified (Supplementary table 7). This information has been added to the manuscript section 4.7.

5) Was IL-6 measured? Its seems to have been but I cannot find it in the test…How does that compare between both types of ARDS? Some studies found this biomarker elevated in COVID19 (JAMA Intern Med 2020; 180: 1152–54. Intensive Care Med 2020; 46: 846–48) but not all of them (Am J Physiol Regul Integr Comp Physiol2021; 320: R250–57) Please discuss.

Response:

Yes, IL-6 was measured. Results are available in Supplementary Table 2, sheet "Proteomics", Row 184.

Below we have copied the relevant information from that Excel sheet, along with a boxplot of the protein levels in the two groups for convenience. As is evident from the table and figure, the IL-6 was not significantly different between the two ARDS groups. IL-6 receptor subunit alpha (IL-6RA) was significantly higher in COVID-19 ARDS and is mentioned in the results section 2.3. We have edited this section to add the information on the IL-6 protein.

"Notably, in our study IL-6 levels were comparable between the two ARDS groups while its receptor IL-6RA was higher in COVID-19 ARDS compared to bacterial sepsis-induced ARDS."

Protein	estimate	std_error	statistic	fold_change	p_value	adj_p	OlinkID	UniProt
IL6	-0.645001	0.638128	-1.01077	-0.645001	0.315874	0.45914	OID00390	P05231

Reviewer Figure: IL-6 levels across the two ARDS groups.

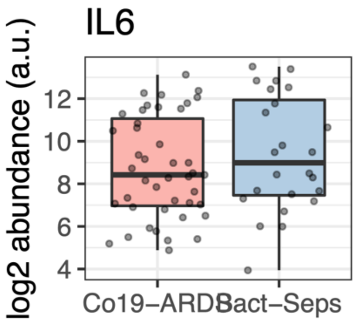

Reviewer #2:

1. Please confirm whether it is serum or plasma. In the section of 4.3 sample handling, it is written as serum, whereas plasma is written elsewhere in the manuscript.

Response: Thanks for catching this mistake. It is a plasma-based study. The sample handling information in section 4.3 was incorrect and has now been updated.

2. The authors stated that samples were stored at 4° C for 1-5 days before being transferred into 80° C freezer. Does this operation affect the expression of metabolites and proteins in the samples? In addition, the samples from COVID-19 ARDS group were collected after 2020, while the earliest samples from bacterial sepsis-induced ARDS group were collected from 2014. What's the impact of the storage on the expression of metabolites. QC data should be provided.

Response: The sample handling information in section 4.3 was incorrect and has been updated. The samples were stored at 4°C for only around 4 hours before being transferred into 80°C freezer.

We do not have QC data for this particular analysis. We relied on prior studies that have performed extensive benchmarking of storage temperatures and durations for the same platforms. Moriya et. al performed an intensive study on the effect of temperature and storage conditions on the plasma metabolomics (4). They reported that the metabolic profiles are largely unaffected by short-term storage at 4°C and long-term storage at -80°C. Similarly, short-term (< 8 hours) storage at 4°C and long-term storage at -80°C has negligible influence on detectable protein levels in plasma measured on the Olink platform, even over years (5,6). We added references to these studies to the respective methods section, which now reads:

"For each participant, whole blood (6-10 mL) was drawn into EDTA-coated blood collection tubes (BD Pharmingen, San Jose, CA). Samples were stored at 4°C and centrifuged within 4 hours of collection to obtain plasma. Plasma was separated and divided into aliquots and kept at -80°C. Previous studies have shown that these conditions ensure stable analytes for the metabolomics, lipidomics, and proteomics platforms used in our study [101–103]."

Reviewer #3: nil

Part III – Minor Issues: Editorial and Data Presentation Modifications

Please use this section for editorial suggestions as well as relatively minor modifications of existing data that would enhance clarity.

Reviewer #1: NA

Reviewer #2:

1. In section 4.8. Differential analysis of molecules, why not to use fold change cutoff to select more robust molecules?

Response: We recognize that dual thresholding using p-values and fold-changes is very common in differential gene expression analysis. Metabolomics changes in particular blood-based metabolomics are usually not as substantial in magnitude, and there is no standardized cut-off that is accepted in the field. Therefore, it is quite common to use a p-value-based threshold in metabolomics studies (some example studies below (7–9)).

2. The authors did not specify if the COVID-19 patients with sepsis were excluded in the Method section?

Response: We have now updated the methods section 4.1 to include this information. Out of the 43 COVID-19 ARDS patients in this study, two patients had a positive blood bacterial culture 72 hrs prior to sample collection. We did not exclude these samples as the clinical research team believed that ARDS was due to progressive COVID-19 infection rather than these secondary infections.

3. The last sentence in the section of introduction: strictly speaking, this is not a large-scale study.

Response: Thank you, this has been edited.

4. "AKI is a sudden reduction in normal kidney function leading to an accumulation of toxic waste products in blood" to "AKI is a sudden reduction in normal kidney function, leading to an accumulation of toxic waste products in blood"

Response: Thank you, this has been edited.

Reviewer #3:

This study provides interesting and valuable data, but the presentation and the data presentation has to be improved to come here well across.

--1st minor but essential point: Data sharing There seem to be four data-sets ready for download at https://doi.org/10.6084/m9.figshare.19775359 This is nice, but could the authors be a bit more explicit in the manuscript what are these data, and, of highest interest to the reader, where do I find the raw data of the study? Furthermore, it would be nice to deposit all original data in a public repository.

Response: We have now updated the shared data repository to contain raw omics profiles. Accordingly, we have also updated the git repository and included a script to process the raw data. In addition, we have improved the description of data in the manuscript.

"Unprocessed as well as preprocessed omics data, along with and clinical data used in this study can be downloaded from https://doi.org/10.6084/m9.figshare.19775359.

All R scripts to process the raw data, analyze the processed data, and generate the tables or figures of this paper are available at https://github.com/krumsieklab/covid-ards-plasma"

--2nd minor but essential point: Insights on the data collection processes some details about the data collection process would be really interesting and should be presented:

A) Which metabolites were difficult to collect? 1,051 lipids, and 266 proteins is fine, but which groups of lipids (e.g. complex lipids such as inflammation mediators) and of proteins (e.g. membrane proteins) are with this experimental set-up difficult to collect?

B) Really good would be that instead of the easy pathway analysis of the metabolites collected the authors educate the reader which blind spots are there, which metabolites can we not follow with this type of analysis? Which of them are probably relevant in this pathophysiology?

Response: Unmeasured molecules are a very important topic in the context of omics platforms with incomplete coverage. There is no agreed-upon consensus or central resource enlisting metabolites, lipids, or proteins that could be measured in a given blood sample. Therefore, a systematic estimation of the negative set of measurements was not attainable.

We recognize this as a limitation of the field as well as our study and accordingly updated the limitations paragraph of the discussion section, describing the issue and several examples of molecules we potentially missed in our measurements. The edited text is copied below for the reviewer's convenience:

"Metabolomics, lipidomics, and proteomics platforms have limits in terms of the coverage of measured molecules, creating the potential for missed associations. For example, our measurement panel did not contain sphingosine-1 phosphate receptor (S1PR), nitric oxide (NO), and nitric oxide synthases (eNOS, iNOS) from Figure 3, angiopoietin 2 (ANGPT2), which has been associated with COVID-19 ARDS-linked vascular necroptosis [92], and proinflammatory lipid groups like prostaglandins [93]. Improvements in measurement technology as well as the integration of data from different platforms will help us generate a more complete picture of ARDS -associated molecular changes in the future."

--Essential: Make clear the added value of the study: We have currently this conclusion "Taken together, our finding of correlations between thrombocytosis (platelet count) and molecules involved in cell adhesion, IL-17, TNF, MAPK signaling pathways imply a coordinated effort of these pathways toward thrombocytosis-mediated coagulopathy

during ARDS"

Now for this conclusion we do not need this study, this is well known from previous publications on the topic and, even more, we can just do a platelet count to see that we have this dangerous ARDS state. Similarly, clinical diagnosis and standard markers allow to easily distinguish between viral or bacterial etiology of the sepsis (starting e.g. from a lymphocyte count).

Response: We believe there might have been a misunderstanding about the structure of our manuscript. The specific sentence quoted above was the final summary of Results section 2.4.2 and only applied to the findings therein**.** The main conclusions and potential implications of our work are described in the Discussion section.

So make a bit more clear to the reader what you really gain from your metabolite and proteomics view: Are there some lipids or proteins visible which were previously not recognized by other groups? Are there interesting and new clinical implications?

Response: We agree that this is important for the paper. The main findings and speculations we provide in the Discussion section are: (1) The role of arginine in long-term sequelae of ARDS, (2) the potential of JAK-STAT inhibitors for severe COVID-19, (3) the role of mitochondrial dysfunction in ARDS**,** and (4) synergy between prothrombotic processes linked to hypercoagulation in ARDS.

Again, we are wondering if these messages got lost to the readers because of a misunderstanding of our paper structure. We would like to refer the referee to the existing discussion section.

To give an exciting example (but maybe just not possible with your current data): is there an early warning metabolite for thromobocytosis, active long before there is thrombocytosis (would be highly interesting)? Do we anyway have metabolites singling out risk patients from safe patients?

I think you are for these points already on the right track singling out markers for kidney injury and for ROS damage / mitochondrial damage, but it would be nice to expand on this and as a general point make a bit more the added value of your network view and multiomics strategy clear compared to a good clinician and simple laboratory tests also of course available.

Response: While our study has a strong clinical component, we regard it as a baseline assessment of the metabolic and biological differences between the different ARDS etiologies. Future studies can build on this work for potential biomarker discovery and treatment options. We did not have a sufficient sample size or design to make this a biomarker study for the prediction of specific conditions. For example, we agree with the

reviewer that an early sign of thrombocytosis before the onset of clinical symptoms would be a very valuable addition to the field; however, our data did not allow us to address this question properly.

The different networks created and the nice views allowing zoom in and out are of course highly appreciated, but sharpening here the conclusions will enhance this paper even further.

Response: We hope that with the clarifications above, this issue is resolved.

References

1. Ziehr DR, Alladina J, Petri CR, Maley JH, Moskowitz A, Medoff BD, et al. Respiratory pathophysiology of mechanically ventilated patients with COVID-19: A cohort study. Am J Respir Crit Care Med. 2020 Jun 15;201(12):1560–4.
2. O'Horo JC, Cerhan JR, Cahn EJ, Bauer PR, Temesgen Z, Ebbert J, et al. Outcomes of COVID-19 With the Mayo Clinic Model of Care and Research. Mayo Clin Proc [Internet]. 2021 Mar 1 [cited 2022 Jun 17];96(3):601–18. Available from: http://www.mayoclinicproceedings.org/article/S0025619620314622/fulltext
3. Toledo JB, Arnold M, Kastenmüller G, Chang R, Baillie RA, Han X, et al. Metabolic network failures in Alzheimer's disease: A biochemical road map. Alzheimer's Dement [Internet]. 2017/03/28. 2017;13(9):965–84. Available from: https://www.ncbi.nlm.nih.gov/pubmed/28341160
4. Moriya T, Satomi Y, Kobayashi H. Intensive determination of storage condition effects on human plasma metabolomics. Metabolomics. 2016 Dec 1;12(12).
5. Shen Q, Björkesten J, Galli J, Ekman D, Broberg J, Nordberg N, et al. Strong impact on plasma protein profiles by precentrifugation delay but not by repeated freeze-thaw cycles, as analyzed using multiplex proximity extension assays. Clin Chem Lab Med [Internet]. 2018 Mar 28 [cited 2022 Jul 20];56(4):582–94. Available from: https://www.degruyter.com/document/doi/10.1515/cclm-2017-0648/html
6. Hebels DGAJ, Georgiadis P, Keun HC, Athersuch TJ, Vineis P, Vermeulen R, et al. Performance in omics analyses of blood samples in long-term storage: Opportunities for the exploitation of existing biobanks in environmental health research. Environ Health Perspect [Internet]. 2013 Apr [cited 2022 Jul 20];121(4):480–7. Available from: http://dx.doi.org/10.1289/ehp.1205657
7. Krumsiek J, Mittelstrass K, Do KT, Stuckler F, Ried J, Adamski J, et al. Gender-specific pathway differences in the human serum metabolome. Metabolomics [Internet]. 2015/10/23. 2015;11(6):1815–33. Available from: https://www.ncbi.nlm.nih.gov/pubmed/26491425
8. Waddington KE, Papadaki A, Coelewij L, Adriani M, Nytrova P, Kubala Havrdova E, et al. Using Serum Metabolomics to Predict Development of Anti-drug

Antibodies in Multiple Sclerosis Patients Treated With IFNβ. Front Immunol. 2020 Jul 17;11:1527.

9. Caterino M, Gelzo M, Sol S, Fedele R, Annunziata A, Calabrese C, et al. Dysregulation of lipid metabolism and pathological inflammation in patients with COVID-19. Sci Rep [Internet]. 2021 Dec 1 [cited 2021 Sep 4];11(1). Available from: https://pubmed.ncbi.nlm.nih.gov/33536486/
